# Iridocorneal endothelial syndrome

**DOI:** 10.3389/fopht.2025.1655669

**Published:** 2025-10-07

**Authors:** Hua Ma, Mingfang Xia, Qing Gu, Lingling Zheng, Shaoping Ha

**Affiliations:** 1 Glaucoma of Ophthalmology, Ningxia Aier Eye Hospital, Yinchuan, Ningxia, China; 2 Aier Academy of Ophthalmology, Central South University, Changsha, Hunan, China; 3 Aier Glaucoma Institute, Hunan Engineering Research Center for Glaucoma with Artificial Intelligence in Diagnosis and Application of New Materials, Changsha Aier Eye Hospital, Changsha, Hunan, China; 4 Eye Center of Xiangya Hospital, Central South University, Changsha, Hunan, China

**Keywords:** iridocorneal endothelial syndrome, progressive iris atrophy, Chandler syndrome, Cogan-Reese syndrome, glaucoma

## Abstract

The iridocorneal endothelial syndrome encompasses a spectrum of ocular disorders predominantly affecting one eye in young to middle-aged women, typically without a familial predisposition. The hallmark feature of iridocorneal endothelial syndrome is the migration of corneal endothelial cells towards the iridocorneal angle and onto the iris. This syndrome comprises three distinct clinical variations: progressive essential atrophy of the iris (including corectopia, iris atrophy, or iris hole), Chandler syndrome (characterized by corneal edema with mild to absent changes in the iris), and Cogan-Reese syndrome (manifesting as nodular pigmented lesions on the front surface of the iris). In cases involving corneal manifestations, such as corneal edema or decompensation, options like Descemet’s stripping automated endothelial keratoplasty and Descemet membrane endothelial keratoplasty may be considered for optimal management. For instance, conditions affecting the iris, such as an iris cavity, multiple pupils, or photophobia, may make femtosecond-assisted keratopigmentation a treatment option. In cases of glaucoma secondary to iridocorneal endothelial syndrome, trabeculectomy with mitomycin C and the implantation of a glaucoma drainage device have been shown to reduce intraocular pressure effectively. At the same time, retrocorneal membrane interception-enhanced peripheral iridectomy has demonstrated significant efficacy.

## 1 Introduction

The iridocorneal endothelial (ICE) syndrome encompasses a collection of ocular disorders, initially proposed by Eagle and Yanoff in 1979 (1). Consequently, ICE mainly occurs unilaterally in young women without a family history (2, 3). While ICE syndrome is relatively uncommon in children, there are few reports that have documented its occurrence in the pediatric population (4–7). The hallmark of ICE syndrome is the migration of corneal endothelial cells towards the iridocorneal angle and onto the iris (8). Thus, ICE is commonly associated with glaucoma, either due to the obstruction of the angle by the proliferating cells or the contraction of their basement membrane over the iris. As such, this results in peripheral traction of the iris and potential development of peripheral anterior synechiae (PAS) (8).

The most significant clinical findings in ICE syndrome are the pathological elements observed in the endothelium, particularly the presence of “the ICE cell” (1, 8). These cells exhibit abnormal enlargement and increased pleomorphism (1, 8, 9). Immunohistochemistry studies have demonstrated the expression of vimentin and cytokeratin, which are typical of epithelial cells (10). Abnormal endothelial cells may increase the risk of corneal edema and decompensation, and they may also migrate posteriorly. Consequently, this leads to the formation of a membrane that extends to cover adjacent structures, such as the iris and trabecular meshwork (11). Moreover, membrane contraction leads to iris changes, iridotrabecular synechiae, corectopia with the pupil being drawn towards the area where the synechiae are most widespread, and to secondary angle-closure glaucoma (11, 12).

Therefore, the ICE syndrome encompasses three clinical variants: progressive essential atrophy of the iris (including corectopia, iris atrophy, or iris hole), Chandler syndrome (characterized by corneal edema with mild to absent iris change), and Cogan-Reese syndrome (manifesting nodular pigmented lesions on the anterior surface of the iris) (13).

## 2 Etiology

The pathogenesis of ICE is not fully understood, and several hypotheses have been proposed. Among the proposed pathogenic mechanisms, the neural crest theory suggests that ICE is associated with the abnormal proliferation of neural crest cells (14). The membrane theory, proposed in 1978, suggests that the initial pathological insult is characterized by corneal endothelial degeneration. Subsequently, this condition progresses to the abnormal over-proliferation of the endothelial membrane surrounding the iridocorneal angle. The contraction of the endothelial membrane, along with the resulting obstruction of the trabecular meshwork, leads to structural alterations in the iris, secondary glaucoma, and ectropion uveae (15). Another pathogenic mechanism, regarded as a viral trigger factor, was proposed when herpes simplex virus DNA was detected in ICE samples (16). In recent study, Findings from multiple sequencing assays uniformly demonstrate that there is no convincing evidence to support viral infection in ICE syndrome. Nevertheless, the transcriptional analysis of ICE cells indicates a suppressed immune response (17).

## 3 Clinical presentation and characteristics

### 3.1 Chandler syndrome

**Figure 1:**
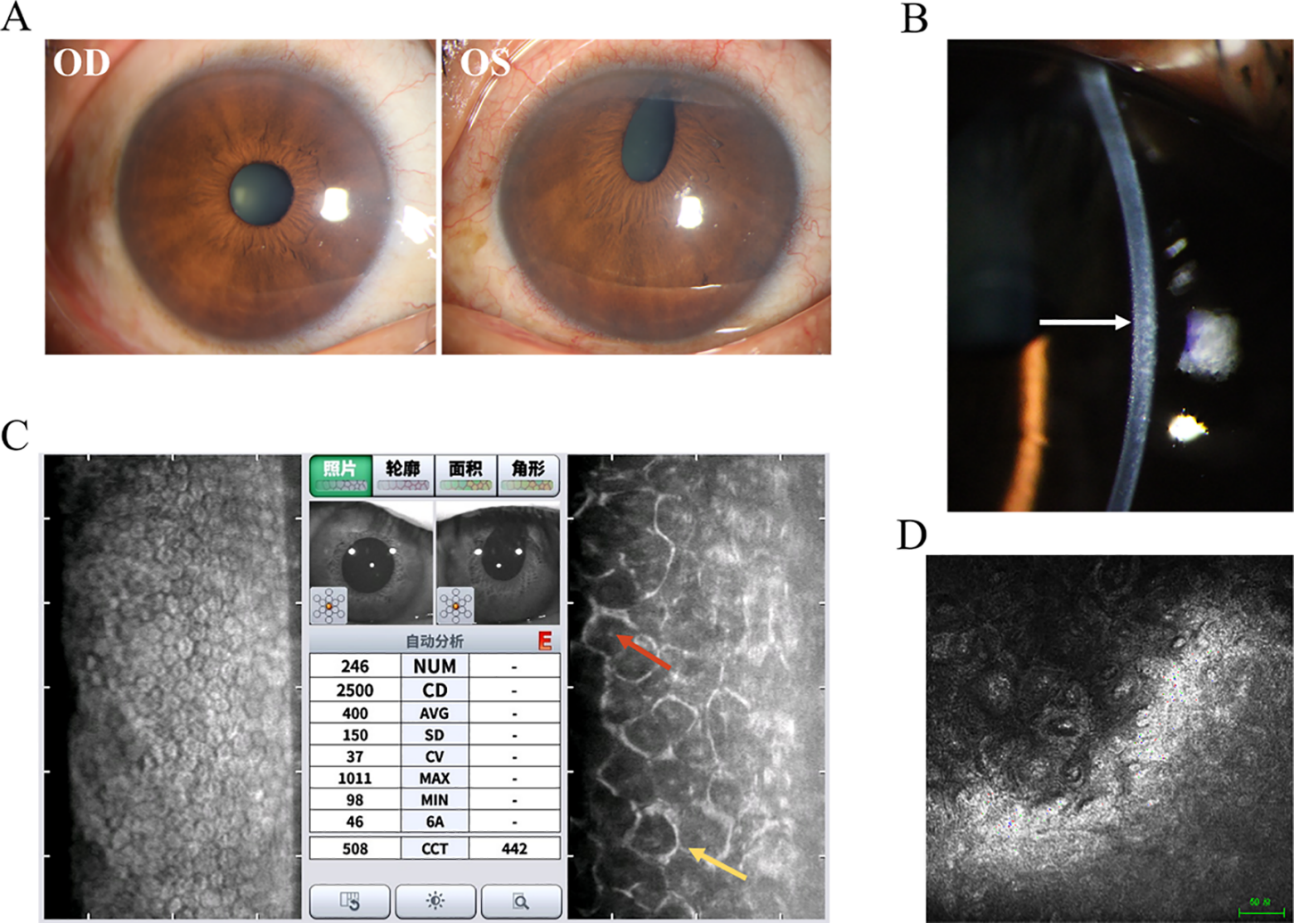
Clinical presentation and ancillary examination findings of Chandler syndrome. **(A)** Slit-lamp image of the left eye demonstrates an obliterated iris with a distorted pupil, while the right eye appears normal. **(B)** Slit-lamp image of the left eye reveals a distinct appearance of the corneal endothelium resembling hammered silver (white arrow). **(C)** Specular microscopy unveils characteristic dark-light reversal pattern (red and yellow arrow) in the corneal endothelial cells, known as "ICE cells". **(D)** Confocal microscopy identifies epithelioid endothelial cells with hyperreflective nuclei.

Chandler syndrome (CS) is defined by unilateral reduced vision, corneal edema, epithelial bullae, a distinctive appearance of the corneal endothelium resembling hammered silver (**Figure 1B**), normal intraocular pressure (IOP) at initial examination, and slight iris atrophy (8). Compared to other types of ICE syndrome, CS typically involves less damage to the iris; however, it is frequently observed that the pupil shows ectopia (**Figure 1A**). When the diagnosis is made at advanced stages, more noticeable iris anomalies may be present, and areas of atrophy can be observed. However, these typically do not result in a complete iris hole (11).

Auxiliary examination plays an irreplaceable role in the clinical diagnosis of ICE. Specular microscopy reveals the presence of ICE cells, characterized by a distinctive pattern of dark-light reversal (9, 18, 19). Notably, the cell surface appears dark rather than light, often exhibiting a centrally located, highly reflective nucleus. In contrast, the intercellular junctions appear light instead of dark (**Figure 1C**). Four different patterns of ICE cell distribution have been identified in the cornea. In “complete ICE,” the normal endothelium is entirely replaced by ICE cells. In “partial ICE (+),” ICE cells replace some of the endothelium, with the remaining portion consisting of small cells. In “partial ICE (–)”, ICE cells replace part of the endothelium. Still, the remaining portion consists of enlarged cells. Lastly, “scattered ICE” refers to individual or small clusters of ICE cells dispersed among normal endothelial cells (18, 20). In the setting of corneal edema, the specular microscope cannot adequately visualize the endothelial changes. However, revealing hyperreflective nuclei, confocal microscopy can still detect epithelioid endothelial cells while maintaining the tissue organization of the corneal endothelium and without the presence of inflammatory cells (21–23) (**Figure 1D**). Confocal microscopy also reveals two distinct patterns of alterations in the epithelioid-like endothelial cells in ICE syndrome: 1. The cells exhibit relatively regular size and shape, resembling normal endothelial cells. However, these are accompanied by a loss of normal hexagonality and prominent, uniform nuclei arranged in a “cobblestone-like” pattern at the center. 2. The endothelial cells display more irregular size and shape, with hyperreflective nuclei of diverse shapes located near the cell boundaries. The stromal nerves seem to exhibit a pseudo-thickening in comparison to the unaffected side (22, 23). Other imaging techniques, such as ultrasound biomicroscopy and anterior-segment optical coherence tomography (AS-OCT), can also be utilized to evaluate the angles in instances of ICE syndrome accompanied by corneal edema (22, 24).

### 3.2 Progressive iris atrophy

**Figure 2:**
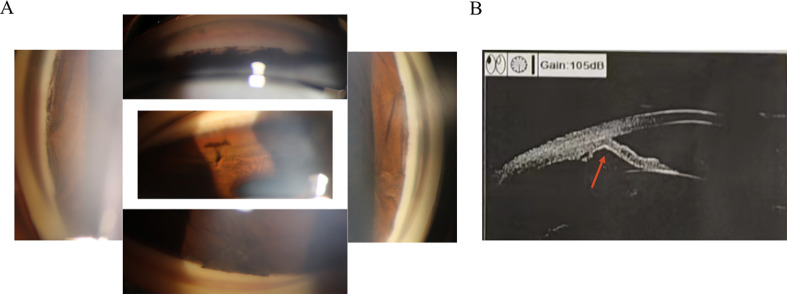
The development of focal peripheral anterior synechiae and causing angle closure of the iridocorneal endothelial syndrome. **(A)** Gonioscopy reveals the presence of local adhesion resulting in closed inspection angle. **(B)** Ultrasound biomicroscopy indicates iris local anterior adhesion (red arrow).

Progressive iris atrophy (PIA) is a disease that progresses slowly over time. Typically, the first clinical occurrence is the development of focal PAS (25). Consequently, proliferation of endothelial cells may play a role in the development of focal adhesions in a previously unobstructed angle, leading to further complications such as increased intraocular pressure and iris retraction (8) (**Figures 2A, B**). The clinical presentation of the cornea can exhibit significant variability. In some cases, a modification in its posterior aspect was demonstrated, while others may additionally manifest corneal edema (26). Changes in the iris are usually detected in the later stages of the disease, marked by the emergence of corectopia and regions of stromal thinning. The pupil is also generally pulled towards a noticeable PAS accompanied by ectropion uveae (15). The iris undergoes stretching on the side opposite to the direction of pupillary distortion, resulting in stromal atrophy and the formation of a “stretch hole” (27). As membranes shrink and lead to progressive synechia formation and pupillary displacement, tension is exerted on the iris. Ultimately, as synechial closure increases, tension on the stroma rises, leading to the breakdown of stromal collagen and the collapse of vessels. Thus, this process results in iris atrophy and the formation of full-thickness holes (11, 27). As mentioned previously, AS-OCT can be utilized for the visualization and accurate documentation of iris atrophy and iridocorneal synechiae caused by ICE syndrome (22).

### 3.3 Cogan-Reese syndrome

The Cogan-Reese syndrome (CRS) is identified by distinctive pigmented nodules present on the iris and a disruption of the typical iris structure. The nodules might initially appear as a small number of delicate, light tan, or yellow bumps on the surface of the iris. The underlying tissue in the area where the nodules are located exhibits a distinct, tangled appearance and has lost its standard features. Iris nodules found in eyes with CRS are usually different, circular or flat, irregular, hyperpigmented spots (28). They are limited to the translucent membrane area and do not appear elsewhere on the iris surface (29). Scheie and Yanoff identified two different kinds of pigmented nodules on the iris in CRS. One type presents small, stalk-like nodules on the surface of the iris. The other type is characterized by a dense appearance within the iris stroma, with a velvety swirling surface and a loss of iris crypts. It is rare for both types of iris lesions to be present in the same eyes (30). Revealing iris nodules composed of polyhedral-to-fusiform melanocytic cells with surface microvilli and long, delicate interweaving dendritic-like processes are observed using transmission electron microscopy. These stromal cells cover long, stout, branching processes that appear to represent the underlying stromal cells of the iris (31). Teekhasaenee and Ritch noted a higher frequency of CRS in Asian eyes, particularly in those with dark brown irides. It is known that darker irides contain a greater amount of melanin pigment granules in the superficial stromal melanocytes compared to lighter irides. Likewise, it appears that a higher concentration of melanin in the iris is associated with an increased likelihood of nodules (29). This is due to alterations in the iris stroma and PAS, resulting in a loss of the typical iris pattern. The iris may appear smoother, with fewer crypts and a reduced pupillary ruff, while circular folds along the pupillary border become less prominent (30). The distortion of pupils is more pronounced in CRS and PIA compared to CS. Typically, the pupils tend to shift towards the location of the membrane and PAS. In CRS, the frequency and intensity of iris atrophy fall between those observed in CS and PIA (8).

## 4 Differential diagnosis

### 4.1 Axenfeld-Rieger syndrome

**Figure 3:**
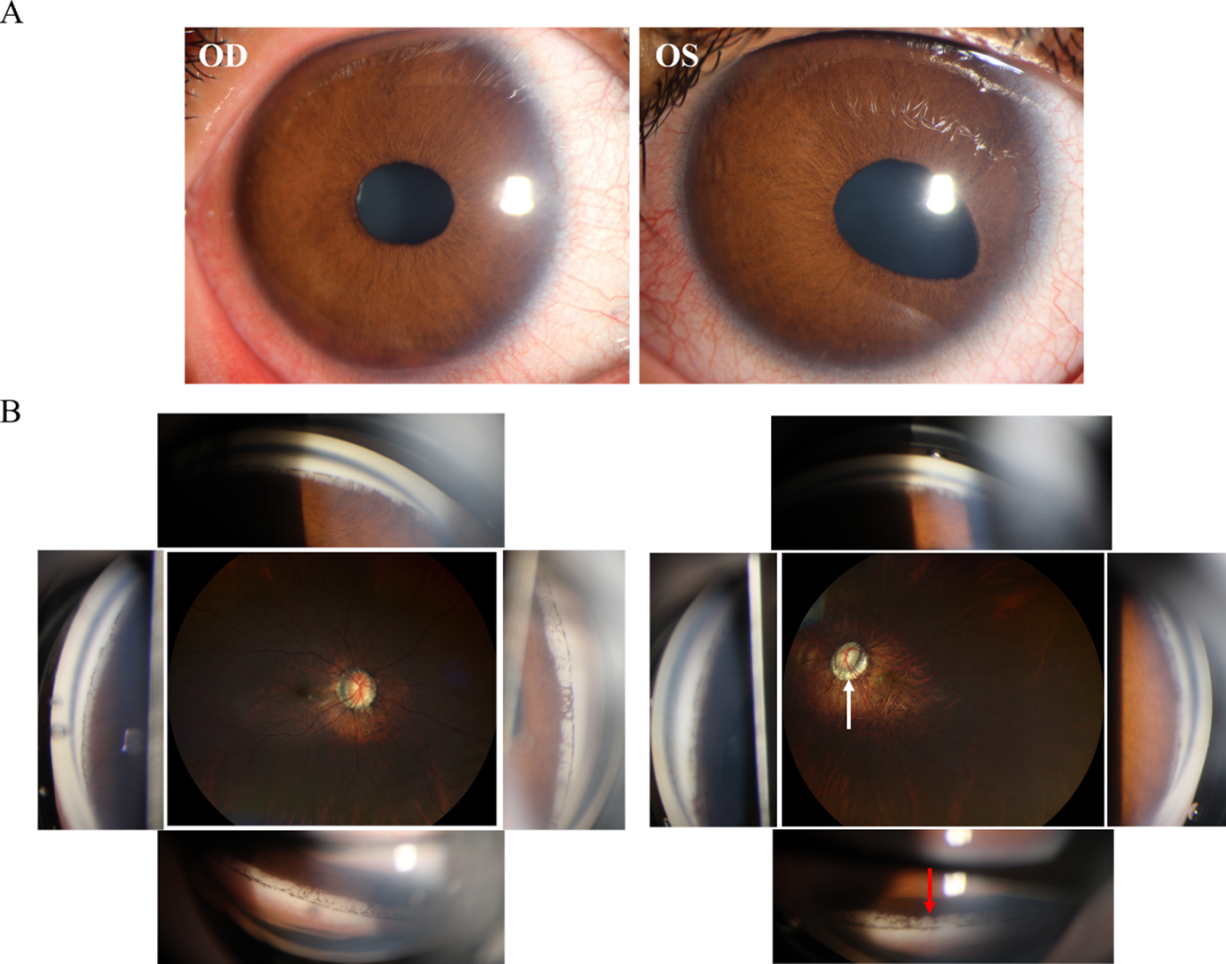
Clinical presentation and ancillary examination findings of Axenfeld-Rieger syndrome (ARS). **(A)** Slit-lamp image of both eyes special the left eye demonstrates an abnormal iris retraction with a displaced pupil. **(B)** Gonioscopy of an eye with ARS reveals distinctive iris strands connected to Schwalbe's line (red arrow).

A hereditary condition affecting both eyes, usually inherited from a parent but can also occur randomly, and involves abnormalities in the iris such as irregular pupil shape, multiple pupils, and connections between the iris and the cornea leading to a corneal posterior embryotoxon (32, 33) (**Figure 3A**). The prominent histopathologic features of ARS include the anterior displacement of Schwalbe’s line and the presence of tissue strands that connect the peripheral iris to the corneal limbus (34). Although ICE and AR syndromes exhibit a single layer of cells resembling endothelial cells. They also exhibit a Descemet-like membrane that stretches from the cornea, through the anterior chamber angle, and onto the front surface of the iris. In ARS, it is believed that the membrane originates from the retention of the primordial endothelial layer lining the anterior chamber during gestation, rather than from the abnormal corneal endothelium acquired after birth, as in ICE syndrome (35). Different from ICE, ARS does not exhibit corneal endothelial changes. A notable distinction between the two conditions is the presence of a posterior embryotoxon with iris strands in ARS (8). In ICE syndrome, instead of iris strands, there may be PAS to the Schwalbe line or beyond, and a posterior embryotoxon is rarely observed (34). Furthermore, the mechanisms of glaucoma vary between the two conditions. A membrane over the trabecular meshwork or PAS in ICE syndrome causes secondary glaucoma. In contrast, maldevelopment of the trabecular meshwork and Schlemm canal, rather than associated iris strands, leads to secondary glaucoma in ARS (32, 34) (**Figure 3B**).

**Figure 4:**
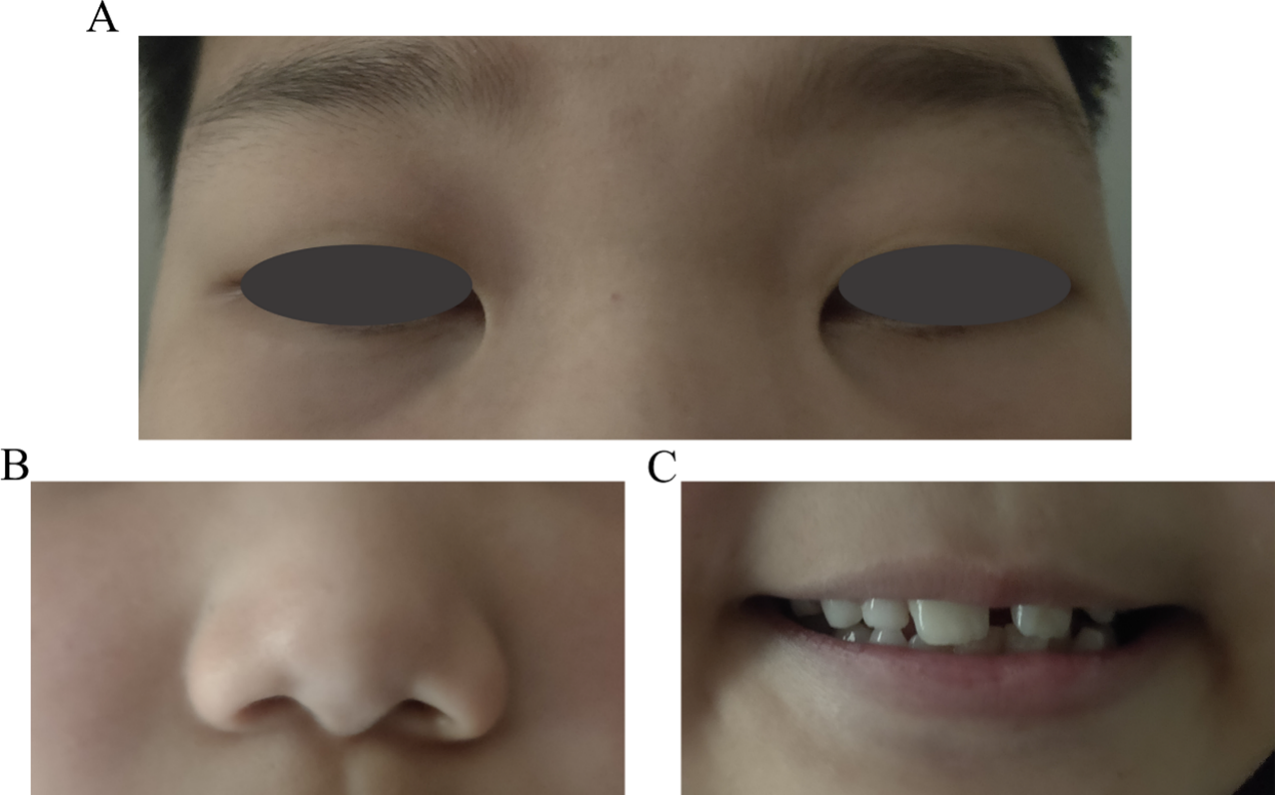
Pediatric patient’s facial characteristics in Axenfeld-Rieger syndrome (ARS). **(A)** The wide-set eyes of facial characteristics in ARS. **(B)** Thin upper lip of facial characteristics in ARS. **(C)** Dental abnormalities often manifest as hypodontia, oligodontia, and microdontia of ARS.

Unlike ICE, ARS in pediatric patients displays a range of systemic observations that indicate abnormal neural crest cell development. Distinctive facial characteristics consist of an underdeveloped upper jaw, wide-set eyes, increased distance between the eyes, a thin upper lip, and a prominent forehead (33, 36) (**Figures 4A–C**). Referred to as maxillary and malar hypoplasia, dental abnormalities often manifest as hypodontia, oligodontia, and microdontia (36) (**Figure 4C**). These are commonly observed characteristics in patients. In addition to other systemic diseases associated with ARS, there may be abnormalities in the gastrointestinal system, excessive umbilical skin, skeletal and cranial abnormalities, as well as cardiac abnormalities (33, 36).

### 4.2 Posterior polymorphous corneal dystrophy

**Figure 5:**
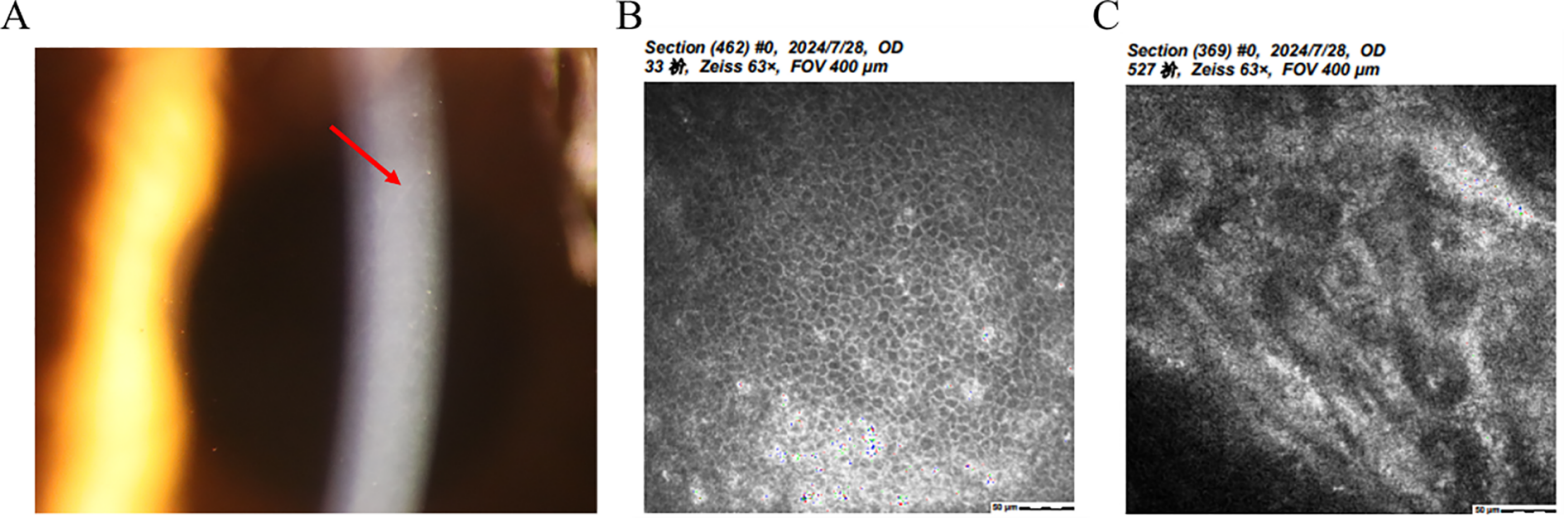
Clinical presentation and ancillary examination findings of Posterior polymorphous corneal dystrophy. **(A)** Slit-lamp image showing a thickened and multilaminar posterior non-banded portion of Descemet’s membrane and epithelium. **(B)** Specular microscopy reveals abnormal reflected signal of corneal epithelium. **(C)** Confocal microscopy demonstrates the presence of characteristic endothelial vesicular structures.

Also known as Schlichting dystrophy, PPCD is a typically bilateral and rare autosomal dominant disorder characterized by epithelial-like endothelial cells and a thickened, multilaminar posterior non-banded portion of Descemet’s membrane (37–40) (**Figure 5A**). Confocal microscopy reveals that the corneal epithelium is in a highly reflective state. At the level of the Descemet's membrane, hyperreflective, placoid or homocentric lesions can be detected, along with hyporeflective, oval or round ones. Additionally, at the level of the endothelial cell layer, hyporeflective, crater-like lesions are evident (40) (**Figures 5B, C**). Clinically, patients may demonstrate clustered vesicles, gray-white plaques, or broad bands with serrated edges on the endothelium (37–39). However, ICE syndrome is typically unilateral, does not have a hereditary pattern, and affects young women more frequently.

### 4.3 Fuchs endothelial corneal dystrophy

**Figure 6:**
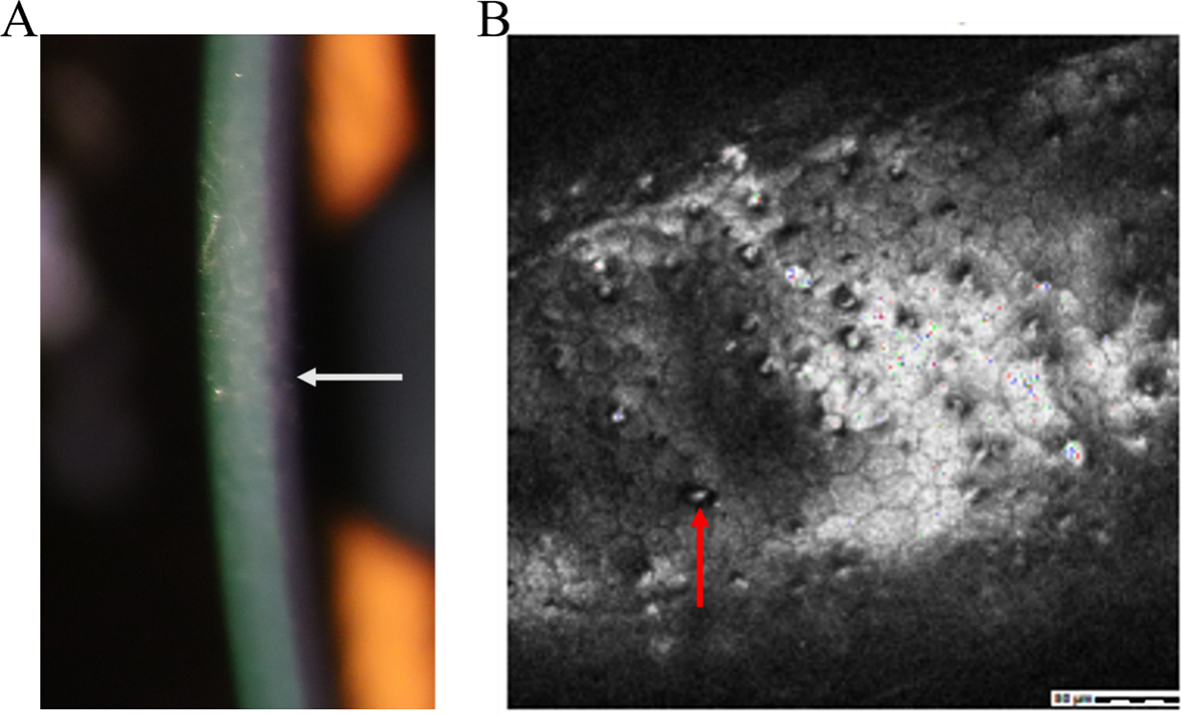
Clinical presentation and ancillary examination findings of Fuchs Endothelial Corneal Dystrophy. **(A)** Slit-lamp image the development of epithelial bullae (white arrow). **(B)** Confocal microscopy of epithelial bullae. abnormal deposits of extracellular matrix components in Descemet membrane (red arrow).

Fuchs Endothelial Corneal Dystrophy is a bilateral disease that progresses slowly and affects both eyes, with a higher prevalence in women aged between 50 and 60 years (41). Initially identified by Ernst Fuchs in 1910, the initial cases showed central clouding of the cornea, decreased corneal sensitivity, and development of epithelial bullae (42) (**Figure 6A**). Upon closer inspection, there is an evident reduction in endothelial cells and the development of guttae, which are abnormal deposits of extracellular matrix components in the Descemet membrane (41–43) (**Figure 6B**).

## 5 Treatment

The management of ICE syndrome can be classified into three primary domains, each addressing distinct facets of the condition. First, it is imperative to effectively address the degeneration of the cornea and any associated complications to preserve visual acuity and mitigate further ocular damage. Secondly, it is crucial to address iris atrophy and its impact on both the aesthetic appearance and visual function of patients with ICE syndrome. This may involve implementing cosmetic interventions to enhance the aesthetic appearance of the affected eye, as well as therapeutic measures to mitigate any visual disturbances resulting from alterations in iris morphology. Finally, it is imperative to manage glaucoma associated with ICE syndrome to prevent further damage to the optic nerve and preserve visual function. This may involve the use of pharmacological agents to reduce intraocular pressure or undergoing surgical interventions.

It is crucial to take a comprehensive approach that encompasses all three primary areas of treatment to effectively manage ICE syndrome and minimize its impact on a patient’s quality of life.

### 5.1 Corneal decompensation and its complications

**Figure 7:**
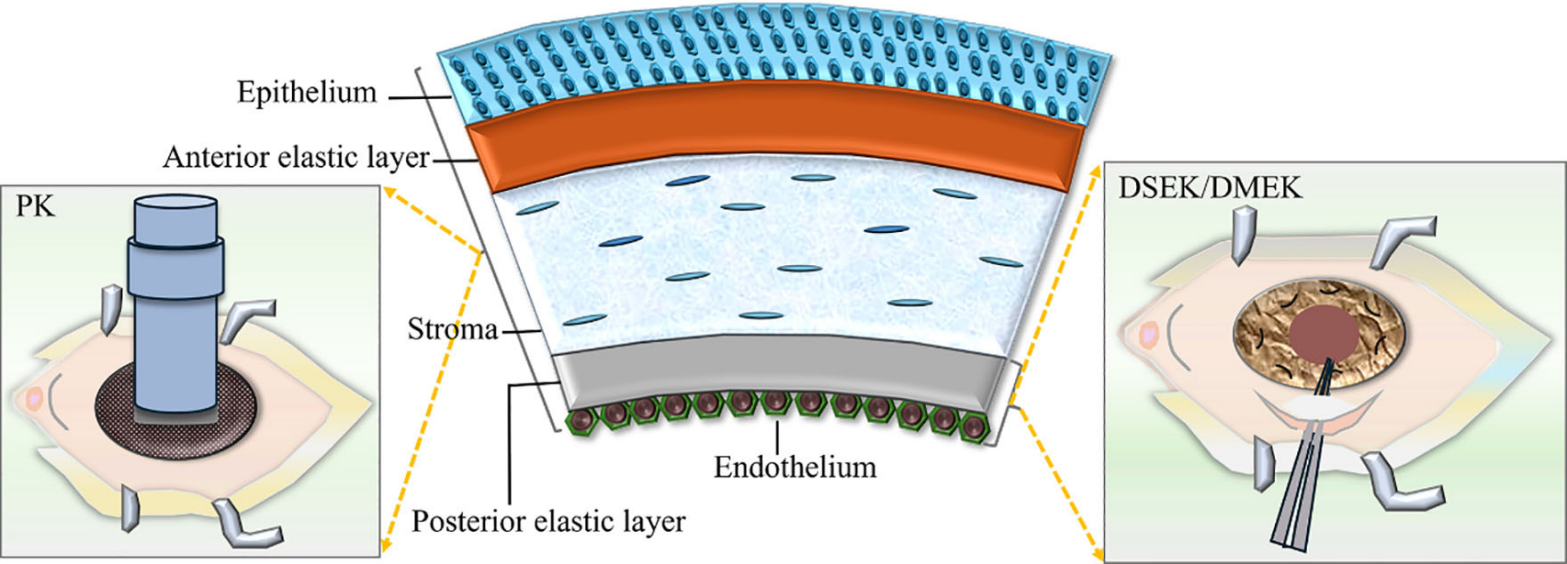
The surgical method and resection range of Penetrating keratoplasty (PK), Descemet stripping endothelial keratoplasty (DSEK) or Descemet membrane endothelial keratoplasty (DMEK).

As for corneal decompensation, penetrating keratoplasty (PK) has demonstrated favorable therapeutic outcomes. However, due to the relatively high rates of rejection and late endothelial failure, repeated surgeries are often indispensable for maintaining corneal clarity (44). When feasible, endothelial keratoplasty techniques are the preferred choices for addressing corneal edema associated with ICE syndrome. Compared to PK, Descemet stripping automated endothelial keratoplasty (DSAEK) and Descemet membrane endothelial keratoplasty (DMEK) are considered more advantageous for ICE syndrome (45). DMEK is generally the preferred option over DSAEK due to its demonstrated superior outcomes, faster recovery, reduced risk of rejection, and minimized postoperative hyperopic shift in refraction. However, DSAEK remains the preferred option for ICE syndrome with significant iris abnormalities or extensive iridocorneal synechiae (46–48). In terms of graft survival, one study indicated that the median graft survival duration for PK was 8.9 years, whereas for DSAEK it was 4.9 years. Nevertheless, due to the insufficient incorporation of data in this research, the median graft survival rate of DMEK lacks statistical significance (45). Previously, DMEK has been demonstrated to have a 0.7% rejection rate, which is lower than PK, along with rapid visual recovery (49). Meanwhile, Chaurasia S and Jing Hong reported that Spokewise Iridotomy, used as an adjunctive surgery for DSAEK in ICE syndrome, resulted in significantly reduced short-term postoperative IOP, decreased AGMs, and PAS recurrence after EK, improved postoperative best corrected visual acuity, and an increased cumulative survival rate of grafts (50, 51). Furthermore, triple procedures (phacoemulsification, intraocular lens insertion, and DSAEK) have shown successful outcomes in CS cases (52). Recently, a case report was published regarding an ICE patient who successfully underwent phacoemulsification, intraocular lens and iris prosthesis implantation into the capsular bag, followed by DSAEK surgery (53). Quek and colleagues found a higher rate of graft failure in essential iris atrophy compared to CS and CRS. This is expected, as eyes with crucial iris atrophy tend to be more aggressive and increase PAS formation (54). (**Figure 7** shows the surgical method and resection range of PK, DSEK, or DMEK).

Patients who have undergone multiple unsuccessful keratoplasties may consider keratoprosthesis as a potential solution for vision restoration (55). Furthermore, cell substitution therapy has demonstrated promising therapeutic outcomes in animal experiments. Notably, this approach involves the injection of cultured human corneal endothelial cells into the anterior chamber, showing promise in eliminating ICE cells by replacing diseased corneal endothelial cells with healthy ones after scraping them (56).

### 5.2 Iris atrophy on appearance and vision

Femtosecond-assisted keratopigmentation is a novel method for improving the appearance of opaque corneas for cosmetic objectives (57). Femtosecond laser technology, which involves intralamellar dissections, can enter the anterior chamber to eliminate degenerated sections of the iris that obstruct the visual pathway. Remarkable postoperative visual acuity outcomes have been reported (13, 58). Pupil ectopia or stroma opacity may lead to issues such as glare and monocular diplopia; a multipiece endocapsular prosthesis for iris reconstruction is an alternative treatment option. And a studies reported that combining cataract extraction with iris reconstruction using a multipiece endocapsular iris prosthesis can help alleviate symptoms and improve appearance (59).

### 5.3 Secondary glaucoma linked to ICE syndrome

**Figure 8:**
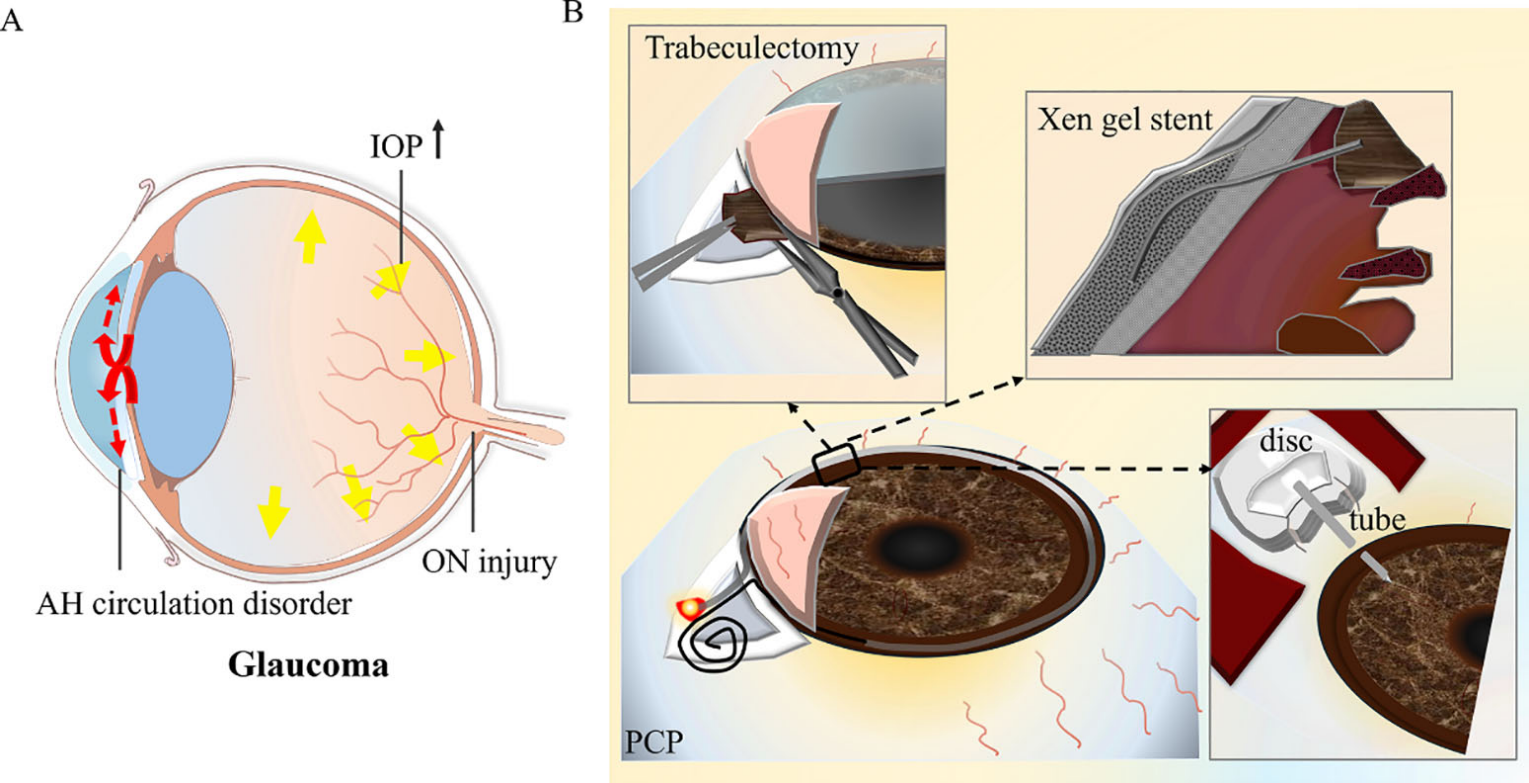
The surgical method and resection range of glaucoma secondary to iridocorneal endothelial syndrome. **(A)** The diagram depicts the optic nerve damage caused by elevated intraocular pressure in glaucoma. **(B)** The diagram indicates the surgical type and primary method. AH, aqueous humor; IOP, intraocular pressure; ON, optical nerve; PCP, penetrating canaloplasty.

The management of glaucoma secondary to iridocorneal endothelial syndrome (GS-ICE) is highly complex, and achieving therapeutic efficiency through simple pharmacological interventions is a significant challenge. In many cases, surgical intervention is frequently warranted. Common surgical modalities include trabeculectomy, implantation of a glaucoma drainage valve, and penetrating canaloplasty (PCP), among others. (**Figure 8** shows the optic nerve damage caused by high IOP in glaucoma (**Figure 8A**) and the surgical type and primary method of GS-ICE (**Figure 8B**)).

Trabeculectomy with mitomycin C or 5-fluorouracil shows a reasonable level of success in the intermediate term for GS-ICE; However, its outcomes are less favorable when compared to juvenile, pigmentary, or primary open-angle glaucoma, likely due to the proliferation of endothelium and the production of abnormal basement membrane within the filtering blebs (18, 60). Glaucoma drainage devices (GDDs) provide an alternative pathway for the outflow of aqueous humor from the anterior chamber, guiding it through a tube to a subconjunctival bleb or to the suprachoroidal space. This approach circumvents the primary concern associated with filtration procedures, namely, the formation of an ICE membrane over the ostium for filtration. It appears to yield a higher success rate for GS-ICE compared to trabeculectomy (61). The implantation of the Ahmed glaucoma valve, Ex-PRESS mini-shunt, or microbypass Xen gel stent (collagen-based gelatin tube) has been demonstrated as a safe and effective approach for the management of GS-ICE (62–64). The primary cause for the failure of aqueous shunt surgery is the blockage of the tube opening by the iris, ICE membrane, or tube displacement. In pseudophakic eyes, we recommend inserting the tube through the ciliary sulcus to reduce the risk of corneal decompensation due to tube-cornea contact and lower the risk of iris damage or iris dialysis in eyes with significant PAS, which is also common in GS-ICE, particularly PIA or CRS (65). Repositioning the tube posterior to the iris may also have the potential to avoid blockage of the inner opening by ICE membrane movement from the iridocorneal angle, as previously reported.

PCP, a novel internal drainage procedure proposed by Yuanbo Liang in 2015, which expands the collapsed Schlemm tube and communicates the anterior chamber and Schlemm tube by excising local trabecular reticulum and peripheral iris, effectively reducing intraocular pressure in ICE syndrome (66, 67). Recently, retrocorneal membrane interception-enhanced PCP has exhibited promising outcomes in the treatment of GS-ICE with open-angle or small PAS for up to one year of follow-up, demonstrating both effectiveness and safety (25).

## 6 Prognosis

The prognosis of the ICE syndrome depends on the disease stage at diagnosis and the presence of secondary complications. Although a variety of treatment modalities are available, corneal surgery may not completely eradicate abnormal endothelial cells. As a result, it is difficult to prevent the progression of PAS or the occurrence of secondary glaucoma (18).

## 7 Conclusion

The ICE syndrome is a complex condition affecting both the cornea and iris, predominantly observed in young females. The hallmark of ICE syndrome is that the corneal endothelial cells migrate towards the iridocorneal angle and onto the iris. Diagnosis is primarily based on the identification of corneal and iris lesions, with auxiliary diagnostic tools including specular microscopy and confocal microscopy. As for CS, Specular microscopy and *in vivo* confocal microscopy may reveal the presence of “ICE cells”. When the cornea is involved, options such as DSAEK and DMEK may be considered for optimal management of conditions like corneal edema or decompensation. In instances where the iris is affected by an iris cavity, multiple pupils, or photophobia, Femtosecond-assisted keratopigmentation may be considered a viable treatment modality. In cases of GS-ICE, trabeculectomy and glaucoma drainage device implantation have been shown to effectively reduce IOP, while PCP, especially retrocorneal membrane interception-enhanced PCP, has demonstrated significant efficacy.

## References

[1] Beganovic AP Vodencarevic AN Halilbasic M Medjedovic A . Iridocorneal endothelial syndrome: case report of essential progressive iris atrophy. Med Arch. (2022) 76:224–8. doi: 10.5455/medarh.2022.76.224-228, PMID: 36200117 PMC9478812

[2] Dubey S Jain K Singh S Mukhejee S . Iridocorneal endothelial syndrome with coexisting macular edema and neurosensory detachment: an unusual case report. J Curr Glaucoma Pract. (2021) 15:149–52. doi: 10.5005/jp-journals-10078-1315, PMID: 35173398 PMC8807940

[3] Malhotra C Seth NG Pandav SS Jain AK Kaushik S Gupta A . Iridocorneal endothelial syndrome: Evaluation of patient demographics and endothelial morphology by *in vivo* confocal microscopy in an Indian cohort. Indian J Ophthalmol. (2019) 67:604–10. doi: 10.4103/ijo.IJO_1237_18, PMID: 31007217 PMC6498939

[4] Aponte EP Ball DC Alward WL . Iridocorneal endothelial syndrome in a 14-year-old male. J Glaucoma. (2016) 25:e115–6. doi: 10.1097/IJG.0000000000000288, PMID: 26035422

[5] Tang W Wang Q Zhang Q Sun S Zhang Y Wu Z . Iridocorneal endothelial syndrome in a Chinese child. Eye Sci. (2013) 28:153–6. doi: 10.3969/j.issn.1000-4432.2013.03.009, PMID: 24579558

[6] Olawoye O Teng CC Liebmann JM Wang FM Ritch R . Iridocorneal endothelial syndrome in a 16-year-old. J Glaucoma. (2011) 20:294–7. doi: 10.1097/IJG.0b013e3181e664b0, PMID: 20616751

[7] Salim S Shields MB Walton D . Iridocorneal endothelial syndrome in a child. J Pediatr Ophthalmol Strabismus. (2006) 43:308–10. doi: 10.3928/01913913-20060901-06, PMID: 17022165

[8] Silva L Najafi A Suwan Y Teekhasaenee C Ritch R . The iridocorneal endothelial syndrome. Surv Ophthalmol. (2018) 63:665–76. doi: 10.1016/j.survophthal.2018.01.001, PMID: 29331589

[9] Levy SG McCartney AC Baghai MH Barrett MC Moss J . Pathology of the iridocorneal-endothelial syndrome. ICE-cell. Invest Ophthalmol Vis Sci. (1995) 36:2592–601. Available online at: https://iovs.arvojournals.org/article.aspx?articleid=2179822 7499082

[10] Hirst LW Bancroft J Yamauchi K Green WR . Immunohistochemical pathology of the corneal endothelium in iridocorneal endothelial syndrome. Invest Ophthalmol Vis Sci. (1995) 36:820–7. Available online at: https://iovs.arvojournals.org/article.aspx?articleid=2161231, PMID: 7706030

[11] Azari AA Rezaei Kanavi M Thompson MJ Altaweel MM Potter HD Albert DM . Iridocorneal endothelial syndrome. JAMA Ophthalmol. (2014) 132:56. doi: 10.1001/jamaophthalmol.2013.247, PMID: 24407828

[12] Bourne WM Brubaker RF . Decreased endothelial permeability in the iridocorneal endothelial syndrome. Ophthalmology. (1982) 89:591–5. doi: 10.1016/s0161-6420(82)34745-4, PMID: 7122039

[13] Walkden A Au L . Iridocorneal endothelial syndrome: clinical perspectives. Clin Ophthalmol. (2018) 12:657–64. doi: 10.2147/OPTH.S143132, PMID: 29670326 PMC5898599

[14] Bahn CF Falls HF Varley GA Meyer RF Edelhauser HF Bourne WM . Classification of corneal endothelial disorders based on neural crest origin. Ophthalmology. (1984) 91:558–63. doi: 10.1016/s0161-6420(84)34249-x, PMID: 6462621

[15] Campbell DG Shields MB Smith TR . The corneal endothelium and the spectrum of essential iris atrophy. Am J Ophthalmol. (1978) 86:317–24. doi: 10.1016/0002-9394(78)90232-5, PMID: 717494

[16] Li F Liu Y Sun Y Zhang X . Etiological mechanism of iridocorneal endothelial (ICE) syndrome may involve infection of herpes simplex virus (HSV) and integration of viral genes into human genome. Med Hypotheses. (2018) 110:50–2. doi: 10.1016/j.mehy.2017.10.023, PMID: 29317068

[17] Zhang J Yuan B Peng R Zhang P Liu X Qu Y . Etiology of iridocorneal endothelial syndrome: viral infection and immune suppression. Cornea. (2025) 00:1-13. doi: 10.1097/ICO.0000000000003911, PMID: 40526465 PMC12753121

[18] Sacchetti M Mantelli F Marenco M Macchi I Ambrosio O Rama P . Diagnosis and management of iridocorneal endothelial syndrome. BioMed Res Int. (2015) 2015:763093. doi: 10.1155/2015/763093, PMID: 26451377 PMC4588350

[19] Chaurasia S Choudhari NS Mohamed A . Clinical and Specular microscopy characteristics and corelation in Iridocorneal endothelial syndrome without corneal edema. Semin Ophthalmol. (2021) 36:561–8. doi: 10.1080/08820538.2021.1900285, PMID: 33750265

[20] Malhotra C Pandav SS Gupta A Jain AK . Phenotypic heterogeneity of corneal endothelium in iridocorneal endothelial syndrome by *in vivo* confocal microscopy. Cornea. (2014) 33:634–7. doi: 10.1097/ICO.0000000000000122, PMID: 24727634

[21] Chaurasia S Vanathi M . Specular microscopy in clinical practice. Indian J Ophthalmol. (2021) 69:517–24. doi: 10.4103/ijo.IJO_574_20, PMID: 33595465 PMC7942069

[22] Güler Canözer G Tınkır Kayıtmazbatır E Öztürk E Bozkurt Oflaz A Bozkurt B . *In vivo* confocal microscopy and anterior segment optical coherence tomography findings of patients with iridocorneal endothelial syndrome. Turk J Ophthalmol. (2024) 54:170–4. doi: 10.4274/tjo.galenos.2024.78861, PMID: 38864597 PMC11589315

[23] Ratra V . Commentary: *In vivo* confocal microscopy in iridocorneal endothelial syndrome. Indian J Ophthalmol. (2019) 67:610–1. doi: 10.4103/ijo.IJO_154_19, PMID: 31007218 PMC6498914

[24] Ichhpujani P Kaushik S Gupta A Pandav SS . Bilateral Chandler’s syndrome: Uncommon entity diagnosed by ultrasound biomicroscopy and confocal microscopy. Indian J Ophthalmol. (2020) 68:528–9. doi: 10.4103/ijo.IJO_1123_19, PMID: 32057025 PMC7043171

[25] Zhou M Zhu S Li H Ye W Xu S Lin H . Retrocorneal membrane interception enhanced penetrating canaloplasty for patients with open angle glaucoma secondary to ICE syndrome. Int Ophthalmol. (2024) 44:395. doi: 10.1007/s10792-024-03211-9, PMID: 39325075

[26] Lee GA . Management of iridocorneal endothelial syndrome from a corneal and glaucoma perspective. Clin Exp Ophthalmol. (2024) 52:491–2. doi: 10.1111/ceo.14372, PMID: 38426385

[27] Ahluwalia NS Shakya R Parikh D . . doi: 10.1177/11206721211067885, PMID: 34923859

[28] Díaz Barrón A Hervás Hernandis JM Duch-Samper AM . Clinical description of a Cogan-Reese type iridocorneal endothelial syndrome using anterior segment optical coherence tomography and specular microscopy. Arch Soc Esp Oftalmol (Engl Ed). (2020) 95:e72. doi: 10.1016/j.oftal.2020.03.006, PMID: 32576400

[29] Teekhasaenee C Ritch R . Iridocorneal endothelial syndrome in Thai patients: clinical variations. Arch Ophthalmol. (2000) 118:187–92. doi: 10.1001/archopht.118.2.187, PMID: 10676783

[30] Scheie HG Yanoff M . Iris nevus (Cogan-Reese) syndrome. A cause unilateral glaucoma. Arch Ophthalmol. (1975) 93:963–70. doi: 10.1001/archopht.1975.01010020761004, PMID: 1180755

[31] Behera G Nag TC Khokhar SK Sangaraju S . Electron microscopy in Cogan-Reese syndrome. Indian J Ophthalmol. (2022) 70:2666–8. doi: 10.4103/ijo.IJO_2777_21, PMID: 35791196 PMC9426052

[32] Michels K Bohnsack BL . Ophthalmological manifestations of axenfeld-rieger syndrome: current perspectives. Clin Ophthalmol. (2023) 17:819–28. doi: 10.2147/OPTH.S379853, PMID: 36926528 PMC10013571

[33] Khandwala NS Ramappa M Edward DP Mocan MC . Axenfeld-Rieger syndrome in the pediatric population: A review. Taiwan J Ophthalmol. (2023) 13:417–24. doi: 10.4103/tjo.TJO-D-23-00089, PMID: 38249500 PMC10798402

[34] Yu T Dai Z Peng R Xiao G Zhang P Ma S . Axenfeld-Rieger syndrome: a novel histopathologic finding associated with corneal abnormalities. BMC Ophthalmol. (2022) 22:514. doi: 10.1186/s12886-022-02754-8, PMID: 36577962 PMC9798569

[35] Seifi M Walter MA . Axenfeld-rieger syndrome. Clin Genet. (2018) 93:1123–30. doi: 10.1111/cge.13148, PMID: 28972279

[36] Reis LM Maheshwari M Capasso J Atilla H Dudakova L Thompson S . Axenfeld-Rieger syndrome: more than meets the eye. J Med Genet. (2023) 60:368–79. doi: 10.1136/jmg-2022-108646, PMID: 35882526 PMC9912354

[37] Liskova P Hafford-Tear NJ Skalicka P Malinka F Jedlickova J Ďuďáková ĽChecktae . Posterior corneal vesicles are not associated with the genetic variants that cause posterior polymorphous corneal dystrophy. Acta Ophthalmol. (2022) 100:e1426–1426e1430. doi: 10.1111/aos.15114, PMID: 35174971

[38] Del Turco C Pierro L Querques G Gagliardi M Corvi F Manitto MP . Posterior polymorphous corneal dystrophy concomitant to large colloid drusen. Eur J Ophthalmol. (2015) 25:177–9. doi: 10.5301/ejo.5000526, PMID: 25363852

[39] Fernández-Gutiérrez E Fernández-Pérez P Boto-De-Los-Bueis A García-Fernández L Rodríguez-Solana P Solís M . Posterior polymorphous corneal dystrophy in a patient with a novel ZEB1 gene mutation. Int J Mol Sci. (2022) 24:209. doi: 10.3390/ijms24010209, PMID: 36613650 PMC9820445

[40] Gu SF Peng RM Xiao GG Hong J . Imaging features of posterior polymorphous corneal dystrophy observed by *in vivo* confocal microscopy. Zhonghua Yan Ke Za Zhi. (2022) 58:103–11. doi: 10.3760/cma.j.cn112142-20210228-00099, PMID: 35144350

[41] Ong Tone S Kocaba V Böhm M Wylegala A White TL Jurkunas UV . Fuchs endothelial corneal dystrophy: The vicious cycle of Fuchs pathogenesis. Prog Retin Eye Res. (2021) 80:100863. doi: 10.1016/j.preteyeres.2020.100863, PMID: 32438095 PMC7648733

[42] Elhalis H Azizi B Jurkunas UV . Fuchs endothelial corneal dystrophy. Ocul Surf. (2010) 8:173–84. doi: 10.1016/s1542-0124(12)70232-x, PMID: 20964980 PMC3061348

[43] Matthaei M Hribek A Clahsen T Bachmann B Cursiefen C Jun AS . Fuchs endothelial corneal dystrophy: clinical, genetic, pathophysiologic, and therapeutic aspects. Annu Rev Vis Sci. (2019) 5:151–75. doi: 10.1146/annurev-vision-091718-014852, PMID: 31525145

[44] Tóth G Váncsa S Kói T Kormányos K Hegyi P Szentmáry N . Outcomes of penetrating keratoplasty versus lamellar endothelial keratoplasty in iridocorneal endothelial syndrome: A systematic review and meta-analysis. Am J Ophthalmol. (2025) 276:218–29. doi: 10.1016/j.ajo.2025.04.017, PMID: 40258484

[45] Roberts PK Keane M Yang G Chan E Harkin DG McKirdy N . Comparison of penetrating and endothelial keratoplasty in patients with iridocorneal endothelial syndrome: A registry study. Clin Exp Ophthalmol. (2023) 51:663–72. doi: 10.1111/ceo.14283, PMID: 37608637

[46] Fajgenbaum MA Hollick EJ . Descemet stripping endothelial keratoplasty in iridocorneal endothelial syndrome: postoperative complications and long-term outcomes. Cornea. (2015) 34:1252–8. doi: 10.1097/ICO.0000000000000530, PMID: 26203744

[47] Ao M Feng Y Xiao G Xu Y Hong J . Clinical outcome of Descemet stripping automated endothelial keratoplasty in 18 cases with iridocorneal endothelial syndrome. Eye (Lond). (2018) 32:679–86. doi: 10.1038/eye.2017.282, PMID: 29243737 PMC5898862

[48] Ghaznawi N Chen ES . Descemet’s stripping automated endothelial keratoplasty: innovations in surgical technique. Curr Opin Ophthalmol. (2010) 21:283–7. doi: 10.1097/ICU.0b013e32833a8cc9, PMID: 20467318

[49] Saleki M Lee P Thaung C Ashena Z . Descemet’s membrane endothelial keratoplasty in an eye with iridocorneal endothelial syndrome and rare association of corneal ectasia. Ther Adv Ophthalmol. (2025) 17:25158414251343968. doi: 10.1177/25158414251343968, PMID: 40837591 PMC12361739

[50] Chaurasia S Senthil S Choudhari N . Outcomes of Descemet stripping endothelial keratoplasty combined with near total iridectomy in iridocorneal endothelial syndrome. BMJ Case Rep. (2021) 14:e240988. doi: 10.1136/bcr-2020-240988, PMID: 33563680 PMC7875288

[51] Zhang J Peng R Xiao G Wang M Hong J . Spokewise iridotomy combined with Descemet stripping automated endothelial keratoplasty in iridocorneal endothelial syndrome. Front Med (Lausanne). (2023) 10:1187009. doi: 10.3389/fmed.2023.1187009, PMID: 37484858 PMC10357380

[52] Kymionis GD Kontadakis GA Agorogiannis GI Bennett M Angelidou F . Descemet stripping automated endothelial keratoplasty combined with phacoemulsification in Chandler syndrome. Eur J Ophthalmol. (2011) 21:495–7. doi: 10.5301/EJO.2010.6210, PMID: 21218389

[53] Rahimi M Panahi Bazaz M Sharifipour F Hajizadeh M Cheraghian B . Corneal biomechanical changes after Descemet stripping automated endothelial keratoplasty, penetrating keratoplasty, and phacoemulsification. Int Ophthalmol. (2022) 42:3183–90. doi: 10.1007/s10792-022-02318-1, PMID: 35552955

[54] Quek DT Wong CW Wong TT Han SB Htoon HM Ho CL . Graft failure and intraocular pressure control after keratoplasty in iridocorneal endothelial syndrome. Am J Ophthalmol. (2015) 160:422–9.e1. doi: 10.1016/j.ajo.2015.05.024, PMID: 26032193

[55] Phillips DL Goins KM Greiner MA Alward WL Kwon YH Wagoner MD . Boston type 1 keratoprosthesis for iridocorneal endothelial syndromes. Cornea. (2015) 34:1383–6. doi: 10.1097/ICO.0000000000000616, PMID: 26398156

[56] Ng XY Peh G Yam GH Tay HG Mehta JS . Corneal endothelial-like cells derived from induced pluripotent stem cells for cell therapy. Int J Mol Sci. (2023) 24:12433. doi: 10.3390/ijms241512433, PMID: 37569804 PMC10418878

[57] Hasani H Es’haghi A Rafatnia S Alilou S Abolmaali M . Keratopigmentation: a comprehensive review. Eye (Lond). (2020) 34:1039–46. doi: 10.1038/s41433-019-0750-2, PMID: 31896801 PMC7253443

[58] Alió JL Rodriguez AE Toffaha BT Piñero DP Moreno LJ . Femtosecond-assisted keratopigmentation for functional and cosmetic restoration in essential iris atrophy. J Cataract Refract Surg. (2011) 37:1744–7. doi: 10.1016/j.jcrs.2011.08.003, PMID: 21865008

[59] Gour A Tibrewal S Garg A Vohra M Ratna R Sangwan VS . New horizons in aniridia management: Clinical insights and therapeutic advances. Taiwan J Ophthalmol. (2023) 13:467–78. doi: 10.4103/tjo.TJO-D-23-00140, PMID: 38249501 PMC10798387

[60] Chandran P Rao HL Mandal AK Choudhari NS Garudadri CS Senthil S . Outcomes of primary trabeculectomy with mitomycin-C in glaucoma secondary to iridocorneal endothelial syndrome. J Glaucoma. (2016) 25:e652–6. doi: 10.1097/IJG.0000000000000268, PMID: 25943731

[61] Imamoglu S Sevim MS Yıldız HE Vural ET Bardak H Bardak Y . Surgical outcomes of patients with iridocorneal endothelial syndrome: a case series. Int Ophthalmol. (2017) 37:607–13. doi: 10.1007/s10792-016-0317-1, PMID: 27495952

[62] Mao Z Guo X Zhong Y Liu X . Surgical outcomes of Ahmed glaucoma valve implantation in patients with glaucoma secondary to iridocorneal endothelial syndrome. Eye (Lond). (2021) 35:608–15. doi: 10.1038/s41433-020-0912-2, PMID: 32367005 PMC8026968

[63] Hohberger B Welge-Lüen UC Lämmer R . ICE-syndrome: A case report of implantation of a microbypass xen gel stent after DMEK transplantation. J Glaucoma. (2017) 26:e103–103e104. doi: 10.1097/IJG.0000000000000584, PMID: 27841796

[64] Colás-Tomás T López Tizón E . Ex-PRESS mini-shunt implanted in a pregnant patient with iridocorneal endothelial syndrome. Eur J Ophthalmol. (2020) 30:NP25–25NP28. doi: 10.1177/1120672118820508, PMID: 30618278

[65] Sun Y Duan X Fang Y Tang X . Long-term surgical outcomes of combined Ahmed glaucoma valve implantation and phacoemulsification with intraocular lens implantation for patients with glaucoma secondary to iridocorneal endothelial syndrome. BMC Ophthalmol. (2024) 24:476. doi: 10.1186/s12886-024-03740-y, PMID: 39482590 PMC11529484

[66] Zhang S Hu C Cheng H Gu J Samuel K Lin H . Efficacy of bleb-independent penetrating canaloplasty in primary angle-closure glaucoma: one-year results. Acta Ophthalmol. (2022) 100:e213–213e220. doi: 10.1111/aos.14869, PMID: 33880864

[67] Deng Y Zhang S Ye W Gu J Lin H Cheng H . Achieving inner aqueous drain in glaucoma secondary to iridocorneal endothelial syndrome: one year results of penetrating canaloplasty. Am J Ophthalmol. (2022) 243:83–90. doi: 10.1016/j.ajo.2022.07.006, PMID: 35870489

