# Supplementary material for: Iridocorneal endothelial syndrome
Source: Front Ophthalmol (Lausanne). 2025 Oct 7;5:1655669. doi: 10.3389/fopht.2025.1655669 (PMC12537377; doi:10.3389/fopht.2025.1655669)
Supplement: Supplementary file 1 [file Table1.docx]

Table: Clinical features and Imaging characteristics of three subtypes of Iridocorneal Endothelial (ICE) syndrome. AS-OCT: anterior-segment optical coherence tomography. TEM: transmission electron microscopy.

|  | Chandler syndrome | Progressive iris atrophy | Cogan-Reese syndrome |
| --- | --- | --- | --- |
| Clinical features | 1. Cornea:  - Corneal edema. - Epithelial bullae. - Corneal endothelium resembling hammered silver.  1. Iris:  - Slight atrophy. - Corectopia(1). | - Corectopia. - Uveae. - Strongly stromal atrophy. - Iris stretch hole(2, 3). | - Pigmented nodules on the iris(4). - Stromal atrophy (between Chandler syndrome and progressive iris atrophy)(1). |
| Image characteristics | 1. Specular microscopy: ICE cell (dark-light reversal)(5). 2. Confocal microscopy: Epithelioid-like endothelial cells(6). | AS-OCT:   - Iris atrophy. - Iridocorneal synechiae(6). | TEM: Polyhedral-to-fusiform melanocytic cells with surface microvilli and long, delicate interweaving dendritic-like processes(7). |


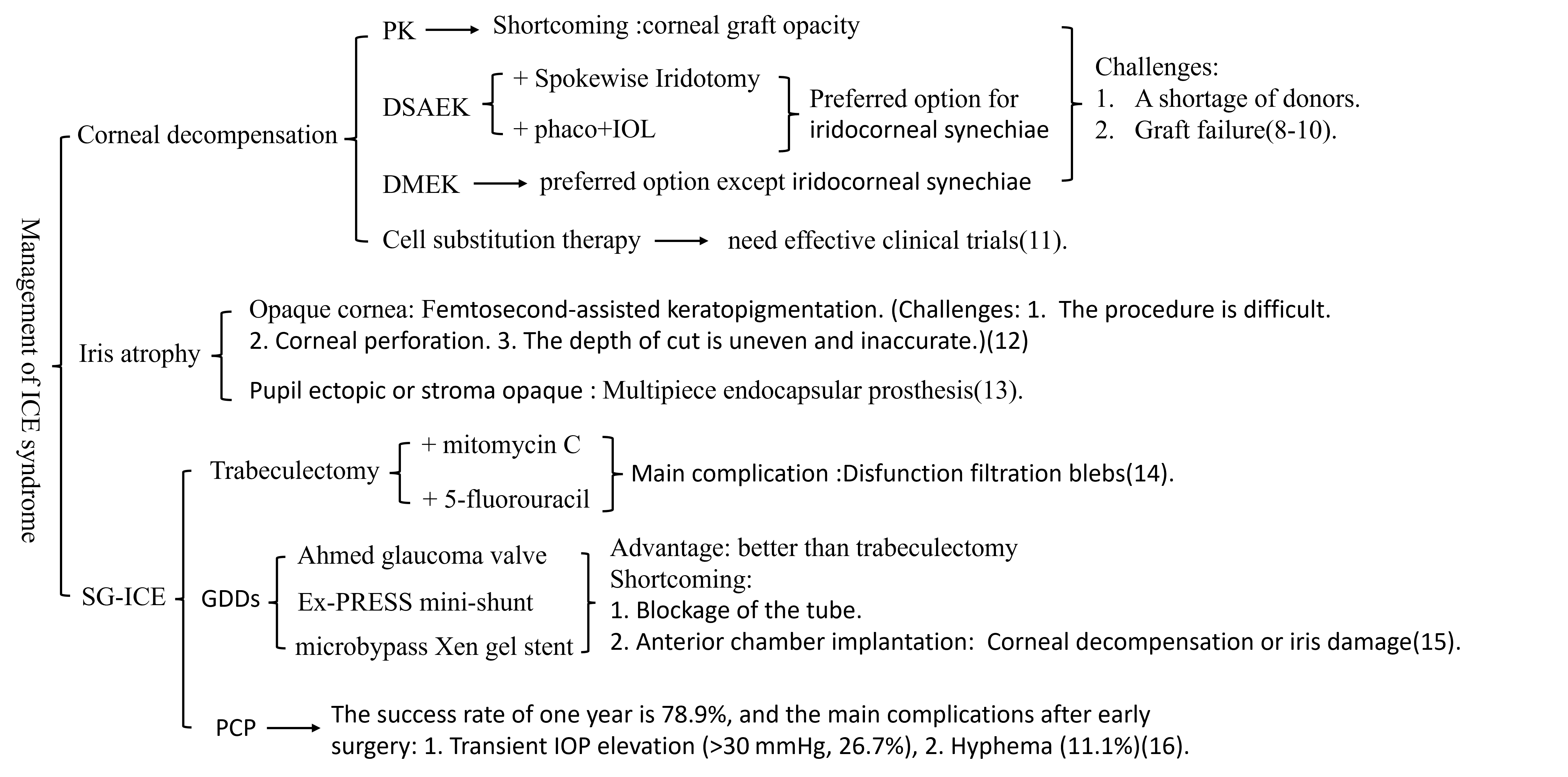
Flowchart: Treatment of Iridocorneal Endothelial syndrome. PK: penetrating keratoplasty. DSAEK: Descemet stripping automated endothelial keratoplasty. DMEK: Descemet membrane endothelial keratoplasty. GDDs: Glaucoma drainage devices. PCP: penetrating canaloplasty.

**References**

1. Silva L, Najafi A, Suwan Y, Teekhasaenee C, Ritch R. The iridocorneal endothelial syndrome. *Surv Ophthalmol*. (2018) 63:665-76. doi: 10.1016/j.survophthal.2018.01.001

2. Campbell DG, Shields MB, Smith TR. The corneal endothelium and the spectrum of essential iris atrophy. *Am J Ophthalmol*. (1978) 86:317-24. doi: 10.1016/0002-9394(78)90232-5

3. Ahluwalia NS, Shakya R, Parikh D. Bilateral laser peripheral iridotomy in a co-occurrence of unilateral iridocorneal endothelial syndrome and chronic angle closure glaucoma. *Eur J Ophthalmol*. (2021) :11206721211067885. doi: 10.1177/11206721211067885

4. Díaz Barrón A, Hervás Hernandis JM, Duch-Samper AM. Clinical description of a Cogan-Reese type iridocorneal endothelial syndrome using anterior segment optical coherence tomography and specular microscopy. *Arch Soc Esp Oftalmol (Engl Ed)*. (2020) 95:e72. doi: 10.1016/j.oftal.2020.03.006

5. Sacchetti M, Mantelli F, Marenco M, Macchi I, Ambrosio O, Rama P. Diagnosis and Management of Iridocorneal Endothelial Syndrome. *Biomed Res Int*. (2015) 2015:763093. doi: 10.1155/2015/763093

6. Güler Canözer G, Tınkır Kayıtmazbatır E, Öztürk E, Bozkurt Oflaz A, Bozkurt B. In Vivo Confocal Microscopy and Anterior Segment Optical Coherence Tomography Findings of Patients with Iridocorneal Endothelial Syndrome. *Turk J Ophthalmol*. (2024) 54:170-4. doi: 10.4274/tjo.galenos.2024.78861

7. Behera G, Nag TC, Khokhar SK, Sangaraju S. Electron microscopy in Cogan-Reese syndrome. *Indian J Ophthalmol*. (2022) 70:2666-8. doi: 10.4103/ijo.IJO_2777_21

8. Fajgenbaum MA, Hollick EJ. Descemet Stripping Endothelial Keratoplasty in Iridocorneal Endothelial Syndrome: Postoperative Complications and Long-Term Outcomes. *Cornea*. (2015) 34:1252-8. doi: 10.1097/ICO.0000000000000530

9. Ao M, Feng Y, Xiao G, Xu Y, Hong J. Clinical outcome of Descemet stripping automated endothelial keratoplasty in 18 cases with iridocorneal endothelial syndrome. *Eye (Lond)*. (2018) 32:679-86. doi: 10.1038/eye.2017.282

10. Ghaznawi N, Chen ES. Descemet's stripping automated endothelial keratoplasty: innovations in surgical technique. Curr Opin Ophthalmol. (2010) 21:283-7. doi: 10.1097/ICU.0b013e32833a8cc9

11. Ng XY, Peh G, Yam GH, Tay HG, Mehta JS. Corneal Endothelial-like Cells Derived from Induced Pluripotent Stem Cells for Cell Therapy. Int J Mol Sci. (2023) 24:12433. doi: 10.3390/ijms241512433

12. Kim JH, Lee D, Hahn TW, Choi SK. New surgical strategy for corneal tattooing using a femtosecond laser. Cornea. (2009) 28:80-4. doi: 10.1097/ICO.0b013e318181a83c

13. Gour A, Tibrewal S, Garg A, Vohra M, Ratna R, Sangwan VS. New horizons in aniridia management: Clinical insights and therapeutic advances. Taiwan J Ophthalmol. (2023) 13:467-78. doi: 10.4103/tjo.TJO-D-23-00140

14. Sacchetti M, Mantelli F, Marenco M, Macchi I, Ambrosio O, Rama P. Diagnosis and Management of Iridocorneal Endothelial Syndrome. Biomed Res Int. (2015) 2015:763093. doi: 10.1155/2015/763093

15. Sun Y, Duan X, Fang Y, Tang X. Long-term surgical outcomes of combined Ahmed glaucoma valve implantation and phacoemulsification with intraocular lens implantation for patients with glaucoma secondary to iridocorneal endothelial syndrome. BMC Ophthalmol. (2024) 24:476. doi: 10.1186/s12886-024-03740-y

16. Zhang S, Hu C, Cheng H, Gu J, Samuel K, Lin H, et al. Efficacy of bleb-independent penetrating canaloplasty in primary angle-closure glaucoma: one-year results. Acta Ophthalmol. (2022) 100:e213-213e220. doi: 10.1111/aos.14869
